# Blood Vessel Invasion Is an Independent Prognostic Factor in Endometrial Endometrioid Carcinoma Compared to Lymph Vessel Invasion and Myometrial Invasion Pattern

**DOI:** 10.3390/cancers16132385

**Published:** 2024-06-28

**Authors:** Senija Eminović, Emina Babarović, Marko Klarić, Dora Fučkar Čupić

**Affiliations:** 1 Department of Pathology and Cytology, Clinical Hospital Center Rijeka, 51000 Rijeka, Croatia; 2 Department of Obstetrics and Gynecology, Clinical Hospital Center Rijeka, 51000 Rijeka, Croatia

**Keywords:** endometrioid endometrial carcinoma, MELF pattern invasion, prognostic value of lymphatic and blood vessel invasion

## Abstract

**Simple Summary:**

Endometrial endometrioid carcinoma (EEC) is generally considered to have a good prognosis. However, a subset of patients die of their disease. For this reason, it is essential to reveal and define all prognostic factors that can guide optimal treatment. Because lymphovascular space invasion (LVSI) is now related to staging, and blood vascular invasion seems to be more important than lymphovascular invasion, it could be important to encourage pathologists to distinguish between the two. The morphologic pattern of myometrial invasion may also be related to biological behavior. Myometrial invasion with an infiltrative pattern is associated with advanced-stage LVSI and recurrence. A specific pattern of myometrial invasion characterized by microcystic, elongated, and fragmented (MELF) glands is known to be associated with LVSI and lymph node metastases. This study investigated the relationship between these three closely related parameters, particularly the importance of blood vessel invasion (BVI), lymphatic vessel invasion (LVI), and MELF invasion as prognostic factors for endometrial cancer. This may be of great importance for the therapeutic approach in EEC.

**Abstract:**

We studied 115 cases of EEC diagnosed on hysterectomy specimens. Double immunohistochemical staining (D2-40/CD31) was performed in all 115 cases to show LVI and BVI on the same slide. MELF pattern invasion was present in 24/115 (21%) cases. MELF-positive tumors had a higher frequency of LVI than MELF-negative tumors (58% and 23%, respectively); the frequency of BVI was twice as high in MELF-positive tumors in comparison to MELF-negative tumors (25% and 12%, respectively). These differences were significant (*p* ˂ 0.0001). All tumors with positive BVI also had a concomitant LVI. The presence of MELF invasion had no impact on overall survival, confirming previous studies. 5-year survival rates were almost equal in cases with negative LVSI and cases with positive isolated LVI (98% vs. 97%). However, in cases where BVI was also present, the 5-year survival rate was significantly lower, 63% (*p* ˂ 0.0001). Furthermore, BVI proved to be an independent prognostic factor for overall survival, disease-free survival, and recurrence in the multivariate analysis. In conclusion, MELF pattern invasion is a good predictor of lymphatic and blood vessel invasion but has no prognostic value. Our results suggest that BVI in EEC has greater clinical value than isolated LVI or myometrial invasion patterns, and the therapeutic approach should be guided by BVI presence. Therefore, we hope this study will promote the routine evaluation of BVI in the context of EEC diagnostic procedures.

## 1. Introduction

Endometrial carcinoma (EC) is the most common malignant disease of the female genital tract worldwide [1]. The most common type of endometrial carcinoma is endometrioid carcinoma (type I), which is usually low grade (grade 1 and grade 2) and generally has a good prognosis. In some patients, however, the prognosis is not so good. The prognosis depends on several factors, including the depth of myometrial invasion, cervical stromal invasion, LVSI, and lymph node involvement. In the last decade, it has been reported that BVI seems to be more important than LVI as a prognostic factor in EEC [2,3,4]. As predictors of hematogenous dissemination BVI, deep myometrial invasion and pattern of myometrial invasion are reported [5,6,7].

In 2020, the European Society of Gynecologic Oncology (ESGO), the European Society of Radiotherapy and Oncology (ESTRO), and the European Society of Pathology (ESP) published guidelines for the management of patients with endometrial cancer. Endometrial cancer has been categorized into five risk groups according to molecular and traditional pathological features such as histopathological type, grade, myometrial invasion, and LVSI. Guidelines are given for cases where the molecular classification is known and for cases where no molecular classification is available. In both, LVSI is recognized as an important prognostic factor [8]. In 2023, the International Federation of Gynecology and Obstetrics (FIGO) presented a new staging system for endometrial cancer [9]. For the first time, LVSI was included in the FIGO staging for endometrial cancer, confirming the importance of this parameter in the prognosis of endometrial cancer.

For many years, a pattern of myometrial invasion has been suggested as a potential prognostic factor [10,11]. This pattern of invasion can be mistaken for LVI because individual cells can be present within glands which are lined by endothelial-like flattened epithelium. MELF pattern invasion has been proposed as a prognostic marker and has been the subject of many research studies. Although in many studies MELF invasion was associated with a higher incidence of LVSI and lymph node metastases, most studies have not demonstrated MELF invasion as an independent prognostic factor [12,13], so the clinical significance of this type of invasion in EEC remains unclear.

Since MELF invasion has been postulated as a good predictor of LVSI in previous studies [14,15] and LVSI is one of the most important factors for disease progression and outcome, in this study we investigated the differential importance of LVI and BVI as prognostic factors associated with MELF invasion. To our knowledge, this is the first study using double D2-40/CD31 immunostaining to investigate the particular importance of BVI and LVI in association with MELF invasion as prognostic factors in endometrial cancer.

## 2. Materials and Methods

The tumor samples analyzed in this retrospective study were obtained from 115 patients with a diagnosis of endometrial endometrioid carcinoma treated with initial hysterectomy and bilateral salpingo-oophorectomy as a standard procedure at the Department of Obstetrics and Gynecology, Clinical Hospital Center Rijeka, Croatia. To avoid selection bias, patients included in the study were selected randomly. Additionally, we defined clear inclusion and exclusion criteria. All patients underwent hysterectomy with lymphadenectomy. Pelvic lymphadenectomy was performed for low-risk carcinomas (low-grade tumors and invasion of less than half the thickness of the myometrium). In the case of high-risk carcinomas (high-grade carcinomas and invasion of more than half the thickness of the myometrium), a para-aortic lymphadenectomy was also performed. All patients had a follow-up of at least 5 years. Since the invasion of the MELF pattern was not detected in intraendometrial tumors, cases without invasion were excluded. A further exclusion criterion was the presence of a serous uterine carcinoma component, a clear cell carcinoma component, or an undifferentiated/undifferentiated carcinoma. All cases with available clinical data and adequate tumor specimens were included in the study. All cases were classified according to the staging system of the International Federation of Gynecology and Obstetrics (FIGO 2009). Adjuvant therapy was agreed on multidisciplinary team meeting: in patients with low-risk endometrial cancer (stage I, low grade, <50% myometrial invasion, LVSI negative), no adjuvant treatment was performed. In low-grade endometrioid adenocarcinoma involving the outer half of the myometrium, brachytherapy is usually given, while if there is associated LVSI, external radiotherapy is administered. In high-grade tumors and advanced stages, chemotherapy was proposed. 

The depth of myometrial invasion was measured between the endometrial–myometrial junction and the deepest point of myometrial invasion and expressed as a percentage of the total thickness of the myometrium. For statistical analysis, myometrial invasion was categorized as <50% or ≥50%. The H&E slides of all 115 cases were examined to assess the presence of MELF invasion. Lymphovascular invasion has been highlighted by double D2-40/CD31 immunohistochemical staining. Clinical data were extracted from patient records. The Ethics Committee of Clinical Hospital Center Rijeka and the Ethics Committee for Biomedical Research Faculty of Medicine Rijeka approved the study.

### 2.1. Immunohistochemistry

Immunohistochemical staining was performed in all 115 cases. A two-color double immunostaining (D2-40/CD31) was performed for differentiated visualization of lymphatic and blood vessels on the same slide. Sections (4 µm) of formalin-fixed, paraffin-embedded tissue blocks were deparaffinized and rehydrated. Two different antibodies were used to detect lymphatic and blood vessels in a single section: the pan-endothelial marker CD31 (monoclonal mouse, clone JC70A, 1:50, DakoCytomation, Glostrup, Denmark) and the lymphatic endothelial marker podoplanin/D2-40 (Monoclonal mouse, clone D2-40,1:100, DakoCytomation, Glostrup, Denmark). Since the best immunohistochemical results for highlighting endothelial cells were obtained with the monoclonal antibody anti-CD31, this was preferred as a marker over anti-CD34, which is expressed in many myometrial smooth muscle cells. A water bath heated to 96 °C was used for antigen retrieval. The slides were immersed in a Tris-EDTA solution with a pH of 9.0 for 15 min. The next steps were performed using the Dako Autostainer Plus (DakoCytomation Colorado Inc, Fort Collins, CO, USA). First, the primary antibody D2-40 was incubated for 30 min. After blocking the endogenous peroxidase with Peroxidase Blocking Solutions (code K8000, DakoCytomation, Glostrup, Denmark) for 5 min, the Flex EnVision+ detection system (Dako, code K8000) was applied for 30 min. DAB chromogen was used for 10 min to develop D2-40 staining. The second primary antibody CD31 was incubated for 30 min. A secondary biotinylated antibody (Dako, code K5005) was applied for 30 min, followed by streptavidin-alkaline phosphatase (Dako, code K5005) for 30 min. Chromogen red (Dako, code K5005) was used to develop CD31 staining. Slides were counterstained with Mayer’s hematoxylin (Dako, code S3309) for 15 s, washed, and mounted with an aqueous medium (Dako, code S3025). Lymphatic vessels and blood vessels were made visible by different colors. The lymphatic endothelium was stained brown, and the endothelium of the blood vessels was stained red. Appropriate controls were used to ensure the quality of the staining.

Endothelium from large vessels was used as an internal positive control for CD31. For D2-40, a normal tonsil was used as a control. For the negative control, the primary antibody was omitted. Invasion of lymphatic vessels was determined based on the presence of tumor cells in a vessel stained with D2-40. The invasion of blood vessels was determined based on the presence of tumor cells in a vessel stained with CD31.

### 2.2. Statistical Analysis

Statistical analysis was performed using MedCalc for Windows, version 17.6. (MedCalc Software, Mariakerke, Belgium). In the first part of the analysis, classical descriptive methods were used. Fisher’s exact test and χ2 test were used to compare differences between nominal variables such as myometrial invasion, grade, stage, and LVSI between categories (patients with MELF pattern invasion versus patients without MELF pattern invasion).

The logistic regression method was used to calculate the odds ratio (OR) for the predictors of recurrence in a univariate and multivariate manner. Eleven patients developed recurrence, and all were categorized as extravaginal recurrences. Two patients had vaginal recurrence, but both had additional multiple metastases (one patient with additional multiple peritoneal metastases and one with lung and brain metastases). Three patients had multiple metastatic foci. All other extravaginal recurrences were metastases in the lungs, peritoneum, soft tissue of the abdominal and chest wall, inguinal and supraclavicular lymph nodes, liver, bone, and retroperitoneum. The median time to development of recurrence was 26.5 months, with a range of 3–72 months. The significance level for all tests was 0.05.

## 3. Results

A total of 115 cases of endometrial carcinomas were involved in the study. The clinicopathological data for the MELF-positive and MELF-negative tumors are summarized in Table 1.

**Figure 1:**
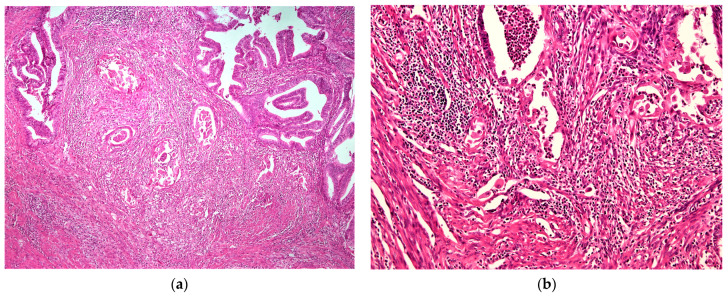
MELF pattern invasion. (**a**) Focus of MELF between conventional infiltrating glands. Microcystic glands lined by flattened, eosinophilic epithelium with luminal inflammatory cells and surrounding myxoid stroma. H&E stain, ×40. (**b**) Glands are fragmented clusters and individual neoplastic cells are admixed with inflammatory cells in the stroma. H&E stain, ×100.

MELF pattern invasion with elongated glands lined by attenuated, eosinophilic, and fragmented neoplastic epithelial cells was observed in 24/115 cases (21%). The surrounding stroma was altered, edematous, fibromyxoid, and contained a prominent inflammatory infiltrate. Occasionally, single neoplastic cells were present in the stroma (Figure 1).

MELF-positive and MELF-negative tumors showed significant differences between the depth of myometrial invasion, tumor grade, and FIGO stage (Table 1). MELF-positive tumors were more likely to invade deeply into the myometrium, with 67% showing deep invasion compared to 27% in MELF-negative tumors (*p* = 0.0006). In addition, tumors with MELF pattern invasion were over twice as likely to reach an advanced stage of ≥IB (79% vs. 35%). The majority of carcinomas in both groups were low grade (grade 1 or grade 2); however, most of the MELF-positive tumors were grade 2 (59%), and MELF-negative tumors were mainly grade 1 (57%). Only 2/24 MELF-positive tumors were grade 3. The difference between the groups, based on the grade, was significant (*p* = 0.044).

**Figure 2:**
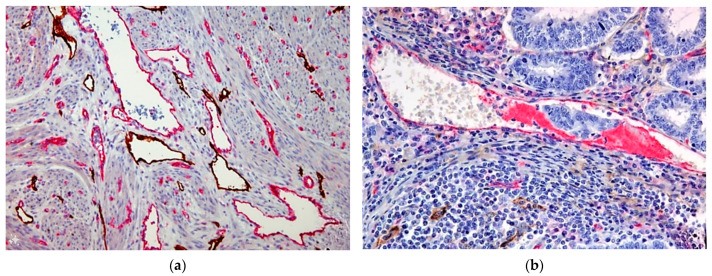
Immunohistochemical double staining results: (**a**) Double immunohistochemical staining for D2-40 and CD31 distinguished the lymphatic vessels (brown) from blood vessels (red). IHC, ×100. (**b**) Peritumoral blood vessel with tumor invasion. IHC, ×200.

LVSI was more frequently observed in MELF-positive than in MELF-negative tumors (83% vs. 35%) (*p* < 0.0001). Next, LVSI components were separately investigated: in MELF-positive tumors, only lymphatic vessel invasion (LVI) was observed in 58% of cases, and blood vessel invasion (BVI) was present in 25% of cases. In MELF-negative cases, LVI was detected in 23% of cases, and BVI in 12% of cases (Figure 2). These results indicate that both lymphatic and blood vessel invasion are more than twice as common in MELF-positive tumors than in the MELF-negative group. 

**Figure 3:**
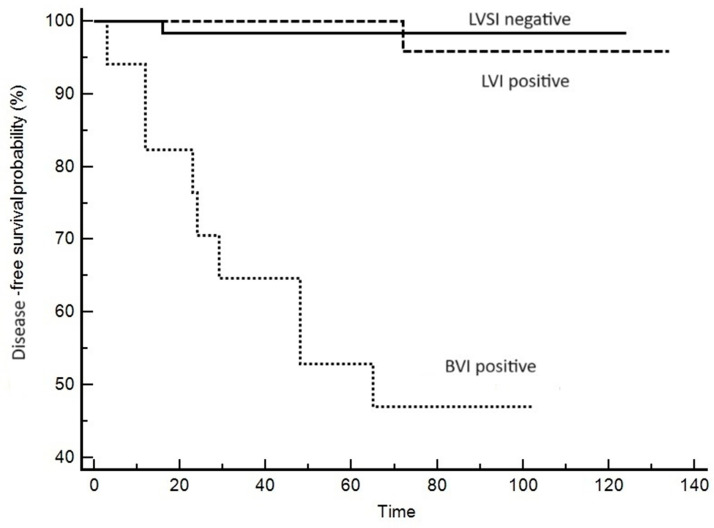
Kaplan–Meier curve of disease-free survival showing the relationship between LVSI, LVI, and BVI. The presence of BVI is associated with shorter disease-free survival.

**Figure 4:**
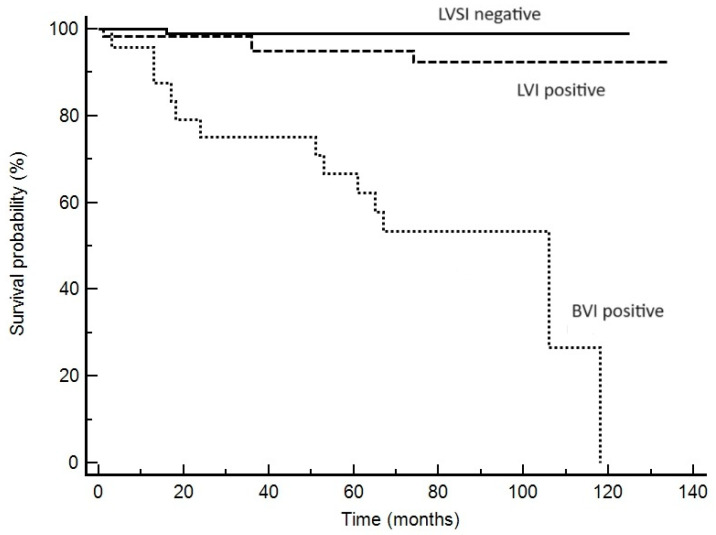
Kaplan–Meier curve of overall survival showing the relationship between LVSI, LVI, and BVI. The presence of BVI is associated with a higher risk of death.

Out of 115 cases, lymph node metastases were present in six cases. Two of them were in the MELF-positive group and four were in the MELF-negative group; this difference, in such a small sample, was not significant. The mean follow-up time was 60 months. Analysis of factors associated with overall survival is shown in Table 2. Disease recurrence occurred in 11 patients, and 12 patients died of the disease. In the univariate analysis, the statistically significant variables affecting disease-free survival and overall survival were tumor grade, FIGO stage, LVSI, and positive lymph nodes. Despite the close association with LVSI, MELF invasion had no impact on recurrence or overall survival in univariate analysis. As shown in Table 2, LVSI-negative tumors and tumors with LVI (without BVI) had almost equal, low risk of mortality. On the contrary, the presence of BVI is associated with a significantly higher risk of recurrence and tumor-related mortality. Kaplan–Meier curves of disease-free survival and overall survival are shown in Figure 3 and Figure 4. In the multivariate Cox regression model, predictors of tumor recurrence included FIGO stage and BVI (Table 3).

## 4. Discussion

This retrospective study analyzed the prognostic significance of three closely related factors in EEC: MELF-type invasion, LVI, and BVI. Our results confirmed that MELF-type invasion, despite being a good predictor of LVSI, has no prognostic significance. BVI is a better prognostic factor than isolated LVI, thus the management of EEC should be based on its presence.

MELF invasion is a histologic pattern of local tumor spread that has been observed in various cancers, including endometrial cancer. MELF pattern invasion in endometrial cancer was described for the first time by Murray et al. in 2003 [16]. While the concept of MELF invasion is not new, it has become increasingly important in recent years as a potential indicator of aggressive tumor behavior and poor clinical outcomes in endometrial cancer [10,11,12,17]. Studies suggest that the presence of MELF invasion in patients with endometrial cancer may be associated with poor prognostic factors such as deep myometrial invasion, LVSI, and lymph node metastasis. While one study suggests that the presence of MELF invasion is a predictor of poorer prognosis in endometrial cancer patients [18], other studies have conflicting results or different interpretations. However, recent studies do not prove a direct association between MELF-type invasion and higher recurrence or worse overall survival [10,11,19,20]. It is important to note that the prognostic significance of MELF invasion in endometrial cancer is still the subject of ongoing research and debate. It has been suggested that MELF invasion may be a predictor of lymphatic invasion, lymph node metastasis, and an overall poorer prognosis.

Lymphovascular space invasion (LVSI) refers to the presence of cancer cells within lymphatic vessels or blood vessels. It is a critical feature in cancer pathology that indicates the potential for cancer cells to spread to other parts of the body via the lymphatic system or bloodstream. LVSI is an important prognostic factor for various types of cancer as its presence suggests a higher risk of metastasis and poorer outcomes. Pathologists carefully examine tissue samples from biopsies or surgical specimens to identify LVSI, as it can influence treatment decisions and patient management. Detection of lymphovascular invasion can guide clinicians in determining the stage of cancer, planning appropriate treatment strategies, and predicting the likelihood of disease recurrence. Treatment options may include more aggressive therapies such as chemotherapy or targeted therapy to reduce the risk of metastasis in cases where LVSI is present.

LVSI in endometrial cancer is an important prognostic factor that can provide valuable information about the aggressiveness of the tumor and the risk of metastasis. The presence of LVSI in endometrial cancer is associated with an increased likelihood of cancer cell spread to lymph nodes. Patients with endometrial cancer and evidence of lymphatic invasion have a higher risk of disease recurrence and poorer survival rates than patients without LVSI, thus identifying LVSI plays a critical role in determining cancer staging, treatment planning, and predicting the likelihood of metastasis in lymph nodes and other distant organs, which may impact treatment decisions and patient outcomes.

Furthermore, the association between BVI and endometrial carcinoma prognosis is a topic of interest in gynecologic oncology research. BVI is considered a poor prognostic factor in endometrial carcinoma as it indicates a higher risk of disease recurrence and metastasis [2,3]. Kymion et al. suggest that BVI is an important factor for cervical stromal invasion in EC. Blood vessel invasion rather than lymphatic vessel invasion is one of the predominant ways through which EC spreads to the cervix [4]. Thus in our study, we explored the impact of BVI on patient outcomes and its association with MELF invasion.

Our results confirm the previously reported strong association between MELF pattern invasion and LVSI in endometrial cancer. However, in this study, we were able to demonstrate a different association between LVI and BVI in MELF-positive tumors. Both LVI and BVI were almost twice as frequent in MELF-positive tumors in comparison to the MELF-negative group. However, despite the close association between LVI and BVI, patients with MELF pattern invasion did not have significantly worse prognosis. These results are consistent with the findings of previous studies.

Concerning LVSI, we divided the tumors into three groups: LVSI-negative, LVI-positive, and BVI-positive. All tumors with BVI had concomitant LVI, and BVI was significantly associated with lower survival and 5-year disease-free survival. LVSI and LVI had similar impacts on disease-free survival and overall survival, which indicates the higher importance of BVI estimation in endometrial carcinoma.

This observation follows other recent findings. Sato et al. revealed that BVI, rather than LVI, is a strong predictor of postoperative recurrence in stage I–III endometrial cancer, probably due to its predisposition to hematogenous metastases The authors concluded that BVI is an important prognostic factor that should be considered in treatment planning and risk stratification for patients with endometrial carcinoma [2].

The 2020 ESGO/ESTRO/ESP guidelines stratify the prognosis of patients with EC by combining the molecular signature of The Cancer Genome ATLAS (TCGA) and pathologic factors, including lymphovascular space invasion (LVSI). Rafone et al. concluded that LVSI has prognostic value independent of TCGA signature, age, and adjuvant treatment [21]. In addition, LVSI was not included in FIGO staging until last year. In 2023, LVSI was recognized for the first time as an important factor for FIGO classification of EC. All these results indicate the importance of LVSI determination in EC. Considering the results of our study and some other findings, BVI assessment is probably more important than LVSI. Markers such as CD31 (stains blood vessels) and D2-40 (specifically stains lymphatic vessels) are not routinely used. Our results indicate the necessity of the determination of BVI.

## 5. Conclusions

Although MELF-type invasion is a strong predictor of LVSI and lymph node metastases, it has no prognostic significance in EEC. Our results suggest that BVI, in contrast to isolated LVI, has greater clinical value as an independent prognostic factor. Therapeutic decisions should probably be based on the presence of BVI. The relationship between BVI and the prognosis of EC is an interesting topic in gynecologic oncology research. BVI is considered a poor prognostic factor in endometrial cancer, as it represents a higher risk of disease recurrence and overall survival. Clinicians need to consider BVI when assessing patients with endometrial cancer for appropriate treatment and follow-up. Pathologists should include BVI presence in the report. We hope that these results will stimulate further research into the prognostic significance of BVI.

## Figures and Tables

**Table 1:** Comparison of clinicopathological features between MELF-negative and MELF-positive tumors.

Features	All	MELF-Negative	MELF-Positive	*p* Value
Patients	115	91 (79%)	24 (21%)	
Age (years) Median (Range)	62 (44–79)	61 (46–76)	63.5 (44–79)	
Myometrial invasion	N (%)	N (%)	N(%)	
<50%	74 (64)	66 (73)	8 (33)	0.0006 ^±^
≥50%	41 (36)	25 (27)	16 (67)	
Grade				
Low grade	102 (89)	80 (88)	22 (92)	0.044 ^¶^
High grade	13 (11)	11 (12)	2 (8)	
Lymph nodes				
Negative	109 (95)	87 (96)	22 (92)	0.603 ^±^
Positive	6 (5)	4 (4)	2 (8)	
FIGO stage				
IA	64 (56)	59 (65)	5 (21)	0.0016 ^¶^
IB	31 (27)	20 (22)	11 (47)	
II	11 (9)	7 (8)	4 (16)	
III and IV	9 (8)	5 (5)	4 (16)	
T-stage				
1a	64 (56)	59 (65)	5 (21)	0.0016 ^¶^
1b	31 (27)	20 (22)	11 (46)	
2	14 (12)	8 (9)	6 (25)	
3 and 4	6 (5)	4 (4)	2 (8)	
LVSI				
negative	63 (55)	59 (65)	4 (17)	0.0001 ^¶^
LVI positive	35 (30)	21 (23)	14 (58)	
LVI + BVI positive	17 (15)	11 (12)	6 (25)	
Site of recurrence				
No recurrence	104 (90)	83 (91)	21 (88)	
Vagina + other site	2 (2)	2 (2)	0 (0)	0.495 ^¶^
Metastasis other locations	9 (8)	6 (7)	3 (12)	
Recurrence				
No	104 (90)	83 (91)	21 (88)	0.695 ^±^
Yes	11 (10)	8 (9)	3 (12)	
Died of disease, N (%)	12 (10)	8 (9)	4 (17)	

^±^ Fisher’s exact test; ^¶^ Chi-squared test.

**Table 2:** The influence of individual variables on the overall survival of patients. In univariate survival analysis using the Kaplan–Meier method, differences between survival curves were determined using the log-rank test.

Factor		N	Died of the Disease	% 5-Year Survival	Log-Rank Test (χ2-Test) *p* Value
Age (years)	≤62	64	5	96	0.268
	>62	51	7	90	
Grade					<0.0001
	Low grade	102	7	90	
	High grade	13	5	68	
FIGO stage	1A	64	1	98	<0.0001
	1B	31	4	94	
	2	11	1	91	
	3 and 4	9	6	55	
pT stage	1a	64	1	98	<0.0001
	1b	31	4	94	
	2	14	3	77	
	3 and 4	6	4	65	
LVSI	Negative	63	1	98	<0.0001
	LVI	35	2	97	
	LVI +BVI	17	9	63	
Lymph nodes	Negative	109	8	96	<0.0001
	Positive	6	4	50	
MELF	Absent	91	8	94.5	0.211
	Present	24	4	87	
Recurrence	No	104	1	99	<0.0001
	Yes	11	11	50	

**Table 3:** Multivariate Cox regression model with disease-free survival.

Predictor	OR	95% CI	*p* Value
Age > 62	3.32	1.14–9.63	0.241
FIGO	3.64	1.55–8.52	0.0029
LVI	0.97	0.61–1.30	0.44
LVI + BVI	9.27	4.47–19.22	<0.0001

## Data Availability

The data presented in this study are available on request from the corresponding author.
